# Transcriptome sequencing and molecular markers discovery in the gonads of *Portunus sanguinolentus*

**DOI:** 10.1038/sdata.2018.131

**Published:** 2018-07-10

**Authors:** Yin Zhang, Guidong Miao, Qingyang Wu, Fan Lin, Cuihong You, Shuqi Wang, Jude Juventus Aweya, Hongyu Ma

**Affiliations:** 1 Guangdong Provincial Key Laboratory of Marine Biotechnology, Shantou University, Shantou 515063, China; 2 STU‑UNIVPM Joint Algal Research Center, Shantou University, Shantou 515063, China

**Keywords:** Animal biotechnology, Transcriptomics, RNA sequencing

## Abstract

Crab culture has gained prominence in the last decade due to the large global market demand for live crabs and crab products. *Portunus sanguinolentus* is one of the economically important crab species in the Indo-Pacific region, with distinct differences in growth and size between male and female crabs, thus, leading to huge difference in their market values. The culture of *P. sanguinolentus* is still in its infancy, with crab supplies heavily dependent on wild catch. In order to unravel the molecular differences between male and female crabs, we generated a comprehensive transcriptomic dataset for *P. sanguinolentus* by sequencing the gonads of both sexes using the Illumina Hiseq 2500 system. Transcriptomes were assembled using Trinity *de novo* assembly followed by annotation. This transcriptomic data set for *P. sanguinolentus* would serve as an important reference data for genomic and genetic studies in this crab and related species.

## Background & Summary

Transcriptome sequencing encompasses several applications including revealing single nucleotide polymorphism (SNP), quantitative trait loci (QTL) and analysis of genes actively expressed in specific tissues or under specific conditions, especially in non-model organisms whose full genome data are unavailable. Successful transcriptome analysis of many economically important aquaculture crab species including *Scylla paramamosain*^1,2^, *Scylla olivacea*^3^, *Portunus trituberculatus*,^4–6^, *Eriocheir sinensis*^7,8^ and *Sinopotamon henanensis*^9^ have been carried out.

Transcriptome data generated from various organs or tissues, including testis^5,10^, ovary^2,11^, accessory sex gland^12^, hepatopancreas^13^ and eyestalk^4^ are usually mined to study the genetic differences between sexes, and to uncover potential sex-related mechanisms. Gonads are the most widely used organs for transcriptome sequencing in reproductive research due to their direct involvement in sexual differentiation, gonadal development and maturation^3,5,12^. Testes are involved in the regulation of androgenic gland hormones and spermatozoa production^13^ in males, whereas ovaries are involved in the production of oocytes and secretion of maturation-related hormones in females^14^.

The crab culture industry has gained importance in the last decade due to high global market demand for live crabs and crab products. Currently, crab culture mainly relies on wild-caught seeds. However, this unregulated crab fishing practice, coupled with various anthropogenic factors, such as overfishing and environmental deterioration, results in a rapid decline in numbers of many economically important crab species^15,16^. *Portunus sanguinolentus* is generally known as the three-spot swimming crab due to the presence of three red to maroon prominent spots on the posterior part of its carapace^17^. It is widely distributed in the Indo-Pacific region, from the east coast of South Africa to Hawaiian waters^18^ and mainly inhabits sandy oceanic habitats to a depth of 30 meters^19,20^. While *P. sanguinolentus* is becoming the main candidate for marine aquaculture due to its high market demand^21^, very limited genetic studies have been carried out on this crab species and its transcriptome information is still unavailable. A recent gonadal transcriptome study conducted during mating embrace on a closely-related portunid species, *P. trituberculatus* revealed several reproduction and gonadal development related genes such as *ADRA1B*, *BAP1*, *ARL3* and *TRPA1* (ref. 6) that might be useful for understanding the molecular mechanism of reproductive development in crabs. However, in genus *Portunus*, the transcriptome resources are only mined in *P. trituberculatus*, which severely limited the genetic studies on other species. So far, no transcriptome resources are available in *P. sanguinolentus*. Thus, gonadal transcriptomic profiles of *P. sanguinolentus* would greatly contribute to molecular marker discovery, help to uncover the regulatory roles of the gonads in portunids, and use as reference for studies in other *Portunus* species.

In the present study, the gonadal transcriptome of *P. sanguinolentus* was sequenced using the Illumina HiSeq 2500 platform. A total of 174,935,588 raw reads and 119,718 unigenes were obtained, with 38,909, 24,641, 31,849, 29,103, 14,937, and 18,406 unigenes annotated to the NR, NT, Swiss-Prot, KEGG, COG, and GO databases, respectively. In addition, a total of 93,196 microsatellites were detected in the unigenes, while 97,364 and 151,626 SNPs were identified in testis and ovary, respectively. This data descriptor provides the first transcriptomic information for *P. sanguinolentus* that is useful for future genomic and genetic studies of this crab and related species. This data would also serve as an important reference for studies on sex differentiation and gonadal maturation mechanisms in marine crabs.

## Methods

### RNA extraction, cDNA library and Illumina sequencing

Total RNA was extracted from the gonads of eight independent *P. sanguinolentus* (four males designated HXX and four females designated HXC, Table 1) using Trizol (Invitrogen, CA, USA) according to the manufacturer’s instructions. NanoDrop^®^ spectrophotometers (Thermo Fisher, MA, USA), Qubit^®^ RNA Assay Kit in Qubit^®^ 3.0 Flurometer (Life Technologies, CA, USA) and RNA Nano 6000 Assay Kit of the Bioanalyzer 2100 system (Agilent Technologies, CA, USA) were used to determine the RNA purity, concentration and integrity values, respectively. Equal amounts of RNA (RNA quality score (RQS) 5.5–7.1 and OD_260/280_ 1.8–2.0) from each individual in the same gender were pooled together as one group, and 1 μg of RNAs from each group used for the library construction. Sequencing libraries were generated using the VAHTS mRNA-seq v2 Library Prep Kit for Illumina® (Vazyme, NR601) following the manufacturer’s recommendations. Firstly, mRNA was purified from the total RNA using poly-A oligo-attached magnetic beads, followed by RNA fragmentation using divalent cations under elevated temperature in Vazyme Frag/Prime Buffer. The cleaved RNA fragments were used for first strand cDNA synthesis using reverse transcriptase and random primers. Second strand cDNA synthesis was subsequently performed using buffer, dNTPs, DNA polymerase I and RNase H. Then, the cDNA fragments were end repaired by the addition of a single ‘A’ base at the 3′-end of each strand, ligated with the special sequencing adapters (Vazyme, N803). The products were purified and size selected using VAHTSTM DNA Clean Beads (Vazyme, N411) in order to obtain the appropriate size (350–450 bp) for sequencing.

The preliminary concentrations of the cDNA libraries were determined using the Qubit® RNA Assay Kit on the Qubit® 3.0 Flurometer, while the insert size was assessed using the Agilent Bioanalyzer 2100 system. Samples with the appropriate insert size were then accurately quantified using qPCR on the Step One Plus Real-Time PCR system (ABI, USA). Next, clustering of the index-coded library samples was performed on a cBot Cluster Generation System (Illumina, USA) according to the manufacturer’s instructions, followed by sequencing on an Illumina Hiseq 2500 platform with 150 bp paired-end module carried out by Vazyme BioTechnologies CO. Ltd (Nanjing, JiangSu, China). The Illumina GA processing pipeline was used for image analysis and base calling.

### *De novo* assembly and functional annotation

In order to obtain high quality sequences for *de novo* assembly analysis, raw reads were filtered by removing: (1) reads containing adapters; (2) reads containing ploy-N (i.e., unrecognized bases) or reads with a ratio greater than 5%; and (3) low-quality reads (number of bases with Q≤10 more than 50% of the entire reads). The clean reads of the two libraries were then assembled into contigs using software Trinity (version: release-20130225, settings: --min_contig_length 150 --CPU 8 --min_kmer_cov 3 --min_glue 3 --bfly_opts '-V 5 --edge-thr=0.1 --stderr'). Three independent software modules including Inchworm, Chrysalis, and Butterfly within Trinity were applied sequentially to process large volumes of RNA-seq reads. First, the Inchworm module assembled the RNA-seq data into unique sequences of transcripts, generating full-length transcripts for a dominant isoform. However, only unique portions of alternatively spliced transcripts (Inchworm contigs) were reported. Next, the Inchworm contigs were assembled into clusters and complete *de Bruijn* graphs were constructed for each cluster using Chrysalis module. Each cluster represents the full transcriptional complexity for a given gene (or sets of genes that shared common sequences). Using the same module, the full read set among disjoint graphs were partitioned. Next, the Butterfly module then processed the individual graphs in parallel, tracing the paths that reads and pairs of reads take within the graph, ultimately reporting full length transcripts for alternatively spliced isoforms, and teasing apart transcripts that correspond to paralogous genes. The resulting sequences from Trinity are called unigenes. Since all samples were of the same species, unigenes from each sample's assembly were further processed with TGICL to cluster assembly sequences, remove redundancy (setting: -l 40 -c 10 -v 25 -O '-repeat_stringency 0.95 -minmatch 35 -minscore 35') and to acquire non-redundant unigenes. Assembly sequences longer than or equal to 200 bp were extracted as unigenes for subsequent analysis. Finally, annotations were assigned to each unigene based on the top hit in BLASTx search against the protein databases, with the non-redundant (NR) protein database at GenBank (http://www.ncbi.nlm.nih.gov) as the highest priority, followed by Swiss-Prot (http://www.expasy.ch/sprot), Kyoto Encyclopedia of Genes and Genomes (KEGG) (http://www.genome.jp/kegg) and Cluster of Orthologous Group (COG) in that priority order. The significant threshold of E-value was set at ≤10^−5^. To further annotate the unigenes, the Blast2GO^22^ program v2.5.0 was employed to obtain their Gene Ontology (GO) annotations based on molecular function, biological process and cellular component features.

### Identification of SSRs and SNPs

The MicroSAtellite (MISA) software (http://pgrc.ipk-gatersleben.de/misa/misa.html) was used to identify SSRs markers in all unigenes, with search criteria as follows: di-nucleotide repeats ≥6, tri-nucleotide to hexa-nucleotide repeats ≥5 and the largest interval between two SSRs was ≤100 bases.

All unigenes were used as reference sequences to detect potential single nucleotide polymorphisms (SNPs) using the SOAPsnp program (http://soap.genomics.org.cn/soapsnp.html)^23^. SNPs sites were predicted based on the different bases at one position in the assembled sequences from the same unigene.

## Data Records

The transcriptome data are available in the NCBI Sequence Read Archive (SRA) database (Data citation 1). Assembly sequence file was uploaded to DDBJ/EMBL/GenBank (Data citation 2). The annotation data (NR, NT, Swiss-Prot, KEGG, COG, and GO database annotations) as well as microsatellites and SNPs were uploaded to figshare (Data Citation 3).

## Technical Validation

A total of 174,935,588 raw reads were generated by Illumina sequencing. After removing the adapter primers as well as low-quality and very short (<50 nt) reads, 167,001,196 clean reads were obtained. Finally, a total of 119,718 unigenes were produced with an average length of 904 nt, which were uploaded to Transcriptome Shotgun Assembly (TSA) project deposited at DDBJ/EMBL/GenBank.

About 43.4% of the unigenes (47,536) were aligned to the protein databases NR at GenBank, Swiss-Prot, KEGG, and COG using BLASTx, while alignment to GO (E-value<10^−5^) was with Blast2GO, and nucleotide database NT (E-value<10^−5^) using BLASTn. A total of 38,909 unigenes were annotated to NR, 24,641 to NT, 31,849 to Swiss-Prot, 29,103 to KEGG, 14,937 to COG, and 18,406 to GO database (Table 2 and Data Citation 3).

**Figure 1:**
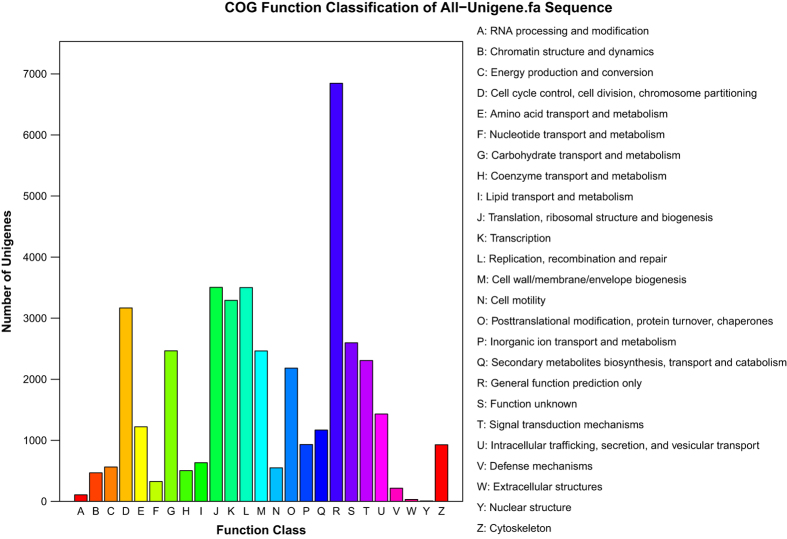
COG functional classification of the *Portunus sanguinolentus* transcriptome.

All unigenes were subjected to functional annotation and classifications analyses. In the COG analysis, 12,617 (14.60%) unigenes were annotated and grouped into 25 COG classifications (Fig. 1 and COG annotation, Data citation 3). The largest cluster was “the general function prediction only (R)”, indicating that the functions of most of the bioinformatics predicted genes had not yet been confirmed by experimentation. The next cluster was “translation, ribosomal structure and biogenesis (J)”, followed by “replication, recombination and repair (L)”, and “cell cycle control, cell division, chromosome partitioning (D)”.

**Figure 2:**
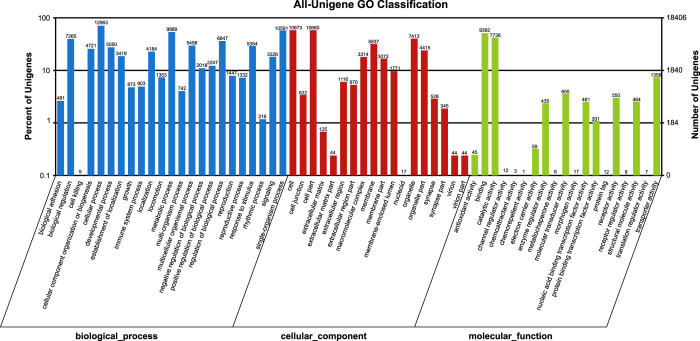
Gene ontology (GO) assignment of assembled unigenes of *Portunus sanguinolentus*. GO classification analysis of Unigenes in All-Unigene. GO functions is showed in X-axis. The right Y-axis shows the number of genes which have the GO function.

For the GO classification, Blast2GO program was first used to obtain the GO annotations followed by the use of WEGO software program to perform the GO functional classification to understand the distribution patterns and functions of the genes at the macro level. A total of 18,406 (15.37%) unigenes were annotated to 60 GO classification terms. In the “biological process” category, majority of the unigenes were represented in cellular process (12,963), single-organism process (10,591), metabolic process (9,889), biological regulation (7,265) and regulation of biological process (6,647). On the other hand, cell (10,673), cell part (10,660), organelle (7,413), membrane (5,837), and organelle part (4,415) were the most represented items in the “cellular component” category, while binding (9,392) and catalytic activity (7,736) were the highest in the molecular function category (Fig. 2, Table 3 and GO annotation, Data citation 3). Further analysis of the GO annotations at different levels revealed that most unigenes were enriched in membrane (Level 1, 1,888 unigenes), protein binding (Level 2, 1,732 unigenes), cytoplasm (Level 5, 1,497 unigenes), nucleus (Level 7, 1,480 unigenes) and integral component of membrane (Level 4, 1,456 unigenes).

Functional classification and pathway assignment based on KEGG analysis showed that metabolic pathways, regulation of actin cytoskeleton, amoebiasis, *Vibrio cholerae* infection and focal adhesion were the top five KEGG pathways (Table 3 and KEGG pathways, Data citation 3). The presence of the *Vibrio cholera* infection pathway among the top five KEGG pathways suggests that virus might have infected the wild crabs used in the study. While this is uncommon due to the ineluctable microbial presence in the open habitat of crabs, it is synonymous with the previous observation where microbial metabolism in diverse environment was among the top 5 KEGG pathways found in gonadal transcriptome profiles of the mud crab, *S. paramamosain*^2,24^.

**Figure 3:**
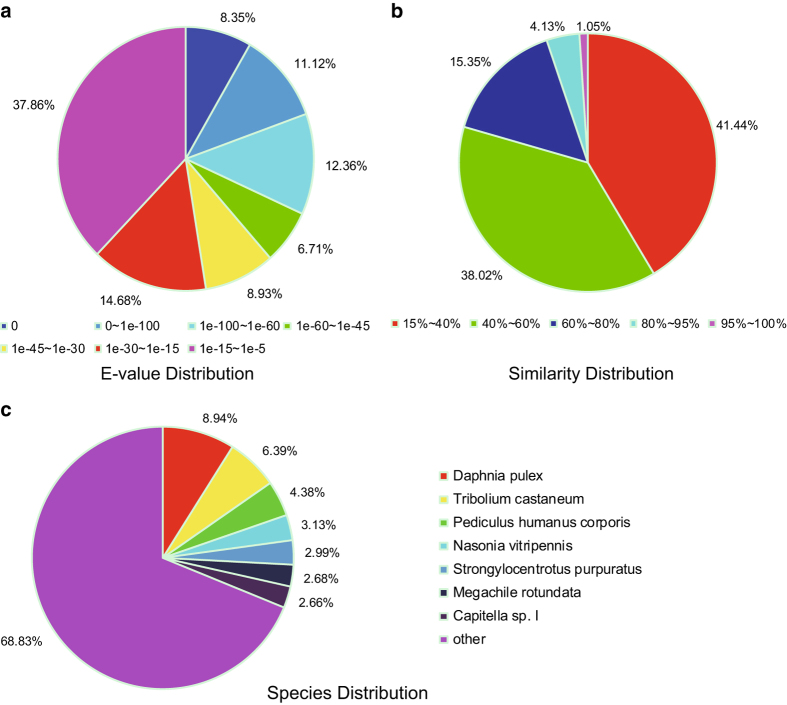
Species that match to the annotated sequences of *Portunus sanguinolentus*. **a**: The E-value distribution of the result of NR annotation. **b**: The similarity distribution of the result of NR annotation. **c**: The species distribution of the result of NR annotation.

About 38.5% of the unigenes exhibited a strong homology with the sequences available in the NR database (E-value <1.0e-45) (Fig. 3a), with 30,958 unigenes matched to the known sequence of 591 species. Most of the unigenes top hit species include the water flea *Daphnia pulex* (3477, 8.94%), red flour beetle *Tribolium castaneum* (2487, 6.39%), body louse *Pediculus humanus corporis* (1703, 4.38%), parasitoid wasp *Nasonia vitripennis* (1219, 3.13%) and purple sea urchin *Strongylocentrotus purpuratus* (1162, 2.99%) (Fig. 3c). Only 5.5% of the unigenes had similarity above 80% (Fig. 3b). (NR annotation, Data citation 3).

In analyzing the differences in gene expression between the two libraries (testis and ovary), the expression of 117,555 unigenes for testis and ovary from the transcriptome data were used. Of these unigenes, 72,517 unigenes were commonly expressed, while 12,503 unigenes were expressed only in ovary and 32,535 unigenes expressed only in testis.

**Figure 4:**
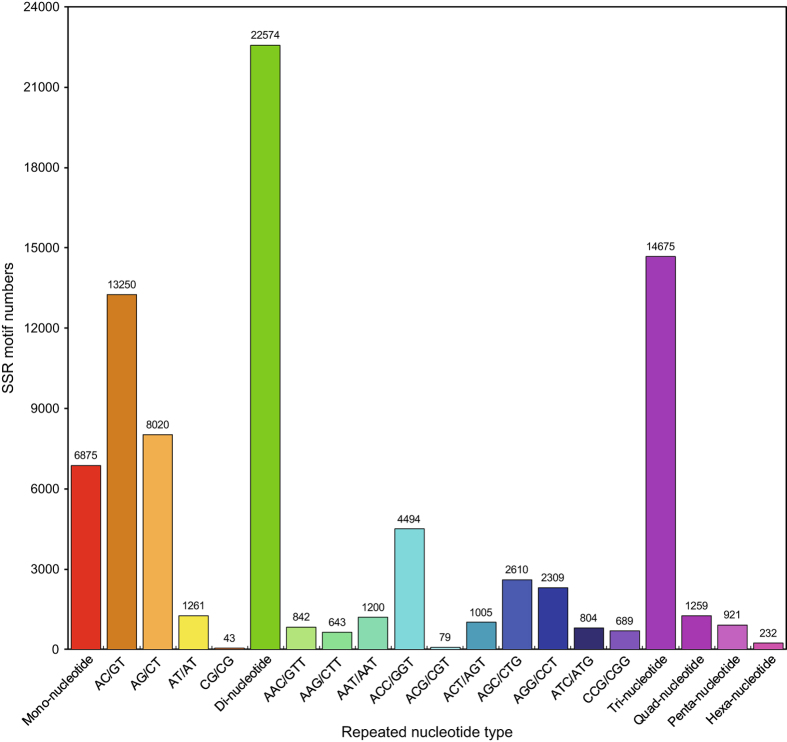
Quantity statistics of SSR classification. The X-axis is the repeat times of repeat units. The Y-axis is the number of SSRs.

**Figure 5:**
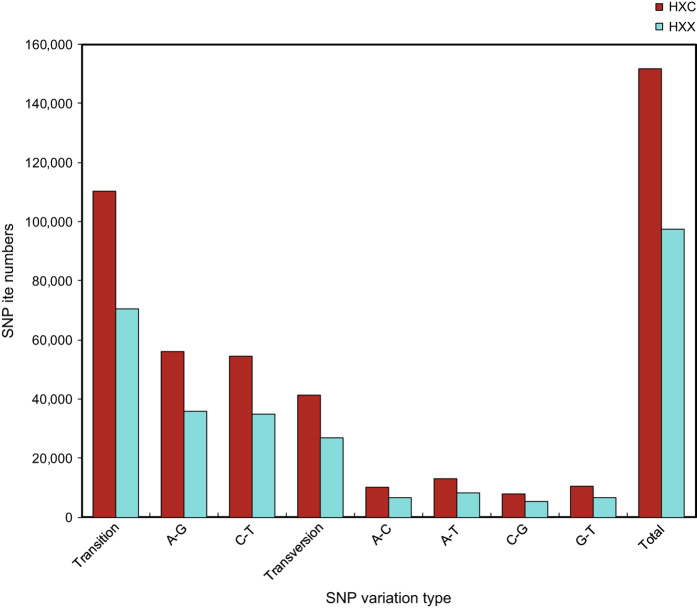
Statistics of SNP number. The X-axis is SNP types, the Y-axis is the number of SNP. HXX represents the male *Portunus sanguinolentus* and HXC represents female *Portunus sanguinolentus*.

The two libraries (testis and ovary) generated a total of 93,196 SSRs (Table 3), with the largest number of SSR motifs being di-nucleotide repeats (22,574) followed by tri-nucleotide repeats (14,675) (Fig. 4 and Microsatellites, Data citation 3). SNP loci were predicted based on the different bases at one position in the assembled sequences from the same unigenes. A total of 97,364 SNPs were detected in the unigenes from the testis library while 151,626 SNPs were found in the ovary library (Table 3). There were about 2.6 times more frequent occurrence of transition type SNPs than transversion in both testis and ovary, with similar numbers of A-G and C-T types being the most abundant transition SNPs in the two groups (Fig. 5 and SNPs, Data citation 3).

The data provided in these experimental datasets are the first report on the transcriptome resources for male and female *P. sanguinolentus*, which includes microsatellite sequences and SNP loci analyzed by Illumina high throughput sequencing technology. These findings are useful for identification of sex-related genes, as well as development of polymorphic genetic markers in *P. sanguinolentus* and other closely related species.

## Additional information

**How to cite this article**: Zhang, Y. *et al*. Transcriptome sequencing and molecular markers discovery in the gonads of *Portunus sanguinolentus*. *Sci. Data* 5:180131 doi: 10.1038/sdata.2018.131 (2018).

**Publisher’s note**: Springer Nature remains neutral with regard to jurisdictional claims in published maps and institutional affiliations.

## Supplementary Material



## Figures and Tables

**Table 1:** Characteristics of transcriptome sequencing project of the *Portunus sanguinolentus*.

Item	Description
Investigation type	Eukaryote transcriptome
Sampling date	13 Mar 2017
Geographic location	23°21′21.47″N, 116°40′38.61″E
Temperature	18–20 ºC
Tissue type	Testes and ovaries
Developmental stage	Adult crabs (ovary stage III-IV; testis stage II-III)
Size	Females: 178.60±60.42 g, Males: 145.05±16.58 g
Sequencing technology	Illumina Hiseq 2500
Assembly	Trinity
Finishing strategy	Contigs
Data accessibility	Bioproject PRJNA415670

**Table 2:** Sequencing, assembly and annotation summary statistics for male (HXX) and female (HXC) *Portunus sanguinolentus*.

Sample	HXC	HXX	All
Total Raw Reads	80,685,590	94,249,998	174,935,588
Total Clean Reads	77,875,810	89,125,386	167,001,196
Total Clean Nucleotides (nt)	11,681,371,500	13,368,807,900	25,050,179,400
Q20 percentage	94.09%	94.80%	
Q30 percentage	86.75	87.95	
N percentage	0.00%	0.00%	
GC percentage	48.97%	50.60%	
contig			
Total Number	138,461	226,875	
Total Length(nt)	59,236,035	76,708,954	
Mean Length(nt)	428	338	
N50	976	547	
unigene			
Total Number	96,434	149,359	119,718
Total Length(nt)	78,041,034	92,887,016	108,264,060
Mean Length(nt)	809	622	904
N50	1830	1318	1712
Total Consensus Sequences	96,434	149,359	119,718
Distinct Clusters	20,187	22,667	30,415
Distinct Singletons	76,247	126,692	89,303
annotation			
NR			38,909
NT			24,641
Swiss-Prot			31,849
KEGG			29,103
COG			14,937
GO			18,406
All annotation unigenes			47,536
SSR			93,196
SNP	151,626	97,364	

**Table 3:** Summary of KEGG pathway and GO annotation for all unigenes.

All unigenes	
	Number of unigenes
Top KEGG pathway	
metabolic pathways	3,609 (12.4%)
regulation of actin cytoskeleton	1,622 (5.57%)
amoebiasis	1,356 (4.66%)
Vibrio cholerae infection	1,222 (4.2%)
focal adhesion	1,205 (4.14%)
Top GO annotation	
biological process	
cellular process	12,963
single-organism process	10,591
metabolic process	9,889
biological regulation	7,265
regulation of biological process	6,647
cellular_component	
cell	10,673
cell part	10,660
organelle	7,413
membrane	5,837
organelle part	4,415
molecular function	
binding	9,392
catalytic activity	7,736
transporter activity	1,358
molecular transducer activity	655
receptor activity	550
Top GO annotation in different levels	
membrane (Level 1)	1,888
protein binding (Level 2)	1,732
cytoplasm (Level 5)	1,497
nucleus (Level 7)	1,480
integral component of membrane (Level 4)	1,456
binding (Level 1)	1,056
ATP binding (Level 8)	972
plasma membrane (Level 4)	935
metabolic process (Level 1)	905
cytosol (Level 7)	876
